# Isolated Respiratory Failure as a Presentation for Early Perinuclear Antineutrophil Cytoplasmic Antibody-Associated Vasculitis

**DOI:** 10.7759/cureus.26698

**Published:** 2022-07-09

**Authors:** Ahmed Mowafy, Asnia Latif, Shruti Jesani, Hasham Saeed

**Affiliations:** 1 Internal Medicine, Trinitas Regional Medical Center RWJBarnabas Health, Elizabeth, USA; 2 Internal Medicine, Rutgers Health/Trinitas Regional Medical Center, Elizabeth, USA

**Keywords:** anca-associated vasculitis, immune hemolytic anemia, lower respiratory tract infection, rituximab therapy, severe respiratory failure

## Abstract

Antineutrophil cytoplasmic antibody (ANCA)-associated vasculitis is an autoimmune inflammatory disease that usually affects the small-sized vasculature, most notably of the lungs and kidneys. One of the challenges of suspecting and diagnosing the condition lies in the insidious and unclear symptoms of presentation. In this case report, we discuss the case of a patient who initially presented with solely unclear pulmonary symptoms, without other organ system dysfunction.

## Introduction

Vasculitis is an inflammatory process that affects blood vessel walls, subsequently causing transmural damage and compromising vessel integrity. The damage affects both vessel integrity and luminal space, leading to vessel ruptures, luminal strictures or obliteration, and thrombosis.

Although the inflammatory process is most commonly a primary disorder due to autoimmune mechanisms, bacterial or viral infections have been shown to induce secondary vasculitis as well.

All vessel calibers are at risk, and vasculitis can be generally classified into small, moderate, or large-vessel vasculitis. Small-vessel vasculitis constitutes the largest group. Overlapping lesions, however, do occur, which explains the wide array of presenting symptoms observed in patients.

Vasculitis is often a serious condition that could lead to severe morbidity and have significant mortality rates if undiagnosed or left untreated. Even with prompt recognition and adequate therapy, mortality rates remain high in vasculitis patients compared to the general population [1].

In this case report, we demonstrate an unclear presentation of vasculitis involving solely the lungs before affecting other systems. The patient’s condition deteriorated quickly before a formal diagnosis could be confirmed, creating the challenge of rapid initiation of immunosuppressive therapy.

We discuss the main difficulties that face the diagnosis of vasculitis and its timely management in an effort to raise awareness about the fairly uncommon condition.

## Case presentation

A 44-year-old female patient with a medical history of asthma and morbid obesity presented to the emergency department (ED) complaining of shortness of breath and dry cough for one week. She noted that her breathing had progressively worsened, preventing her from her activities of daily living. She described her cough as dry, with intermittent scant blood-tinged sputum production. She denied fevers, chills, chest pain, night sweats, history of tuberculosis, recent travel, or sick contacts.

On presentation, the patient was afebrile, body temperature was 97°F, she was tachycardic with a heart rate of 117 beats per minute, tachypneic with a respiratory rate of 32 breaths per minute, and was hypotensive with a blood pressure of 82/52 mmHg. She was also found to be hypoxemic, saturating 72% on room air. The patient was promptly treated with intravenous (IV) normal saline for the hypotension and was placed on 6 L of oxygen via nasal cannula, upon which her oxygen saturation improved to 90%.

Her labs revealed hemoglobin of 9.0 g/dL (12-16 g/dL), with an mean corpuscular volume of 98 fL (80-94 fL), white blood cell of 4.2 (4.8-10.8), serum sodium of 137 mmol/L (136-146 mmol/L), potassium of 3.7 mmol/L (3.6-5.1 mmol/L), chloride of 100 mmol/L (101-111 mmol/L), bicarbonate of 23 mmol/L (22-32 mmol/L), creatinine of 0.62 mg/dL (0.4-1.0 mg/dL), and blood urea nitrogen of 8 mg/dL (8-20 mg/dL). Her inflammatory markers were elevated, with C-reactive protein at 5.3 ng/dL (<1 ng/dL), erythrocyte sedimentation rate of 30 mm/hour (0-20 mm/hour), lactate dehydrogenase of 334 U/L (98-192 U/L), fibrinogen of 550 mg/dL (250-500 mg/dL), and a D-dimer of 424 ng/mL (0-230 ng/mL). Her lactic acid was 1.03 mmol/L (0.5-2.2 mmol/L).

**Figure 1:**
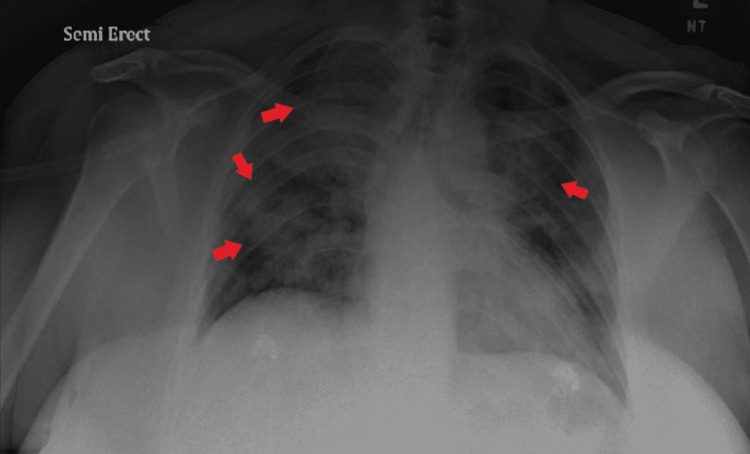
Chest X-ray revealing patchy ground-glass opacities within mid and lower lung fields.

**Figure 2:**
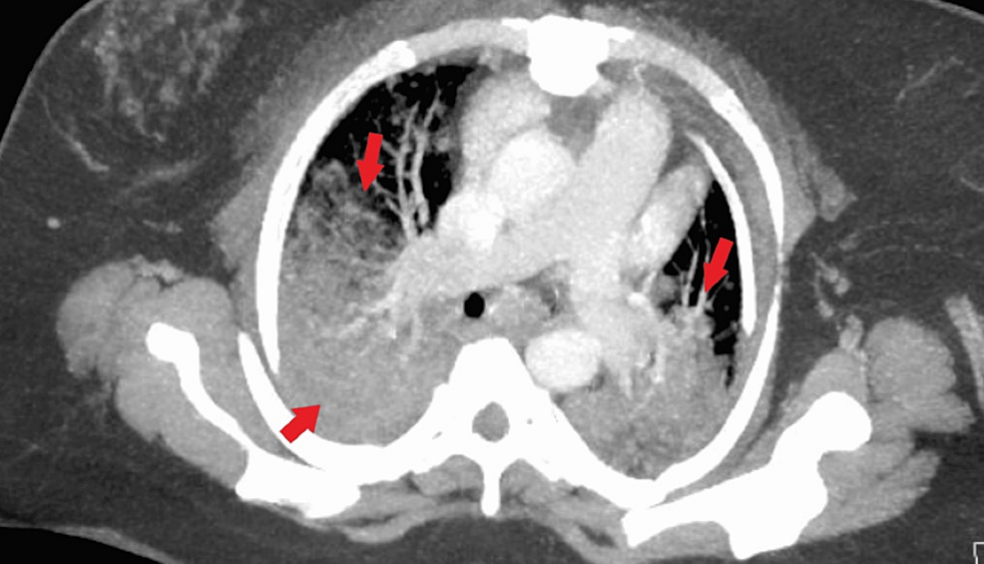
Computed tomography angiogram of the chest revealing no pulmonary embolism but positive for extensive bilateral alveolar consolidation and ground-glass opacification.

**Figure 3:**
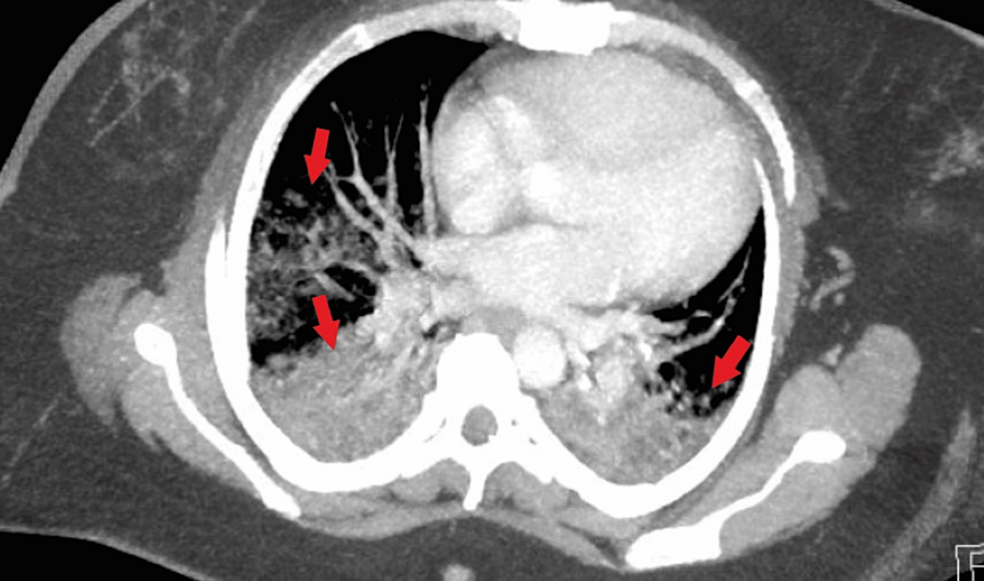
Computed tomography angiogram of the chest revealing extensive bilateral alveolar consolidation and ground-glass opacification.

Her chest X-ray revealed moderate patchy ground-glass opacities within mid and lower lung fields, with no pleural effusions of pneumothorax (Figure 1). Computed tomography angiogram of the chest was obtained to rule out a pulmonary embolism (PE). The scan was negative for PE but demonstrated extensive alveolar consolidation of both upper lobes with numerous air bronchograms, diffuse consolidation of the left lower lobe with lingular sparing, diffuse subsegmental ground-glass opacification throughout the right middle lobe, and dense consolidation of the right lower lobe (Figures 2, 3).

Rapid testing for coronavirus disease 2019 (COVID-19) polymerase chain reaction was negative, pneumococcal and legionella urine antigens were negative, and influenza and respiratory syncytial virus antigens were negative as well.

The patient was admitted to the hospital for severe pneumonia and started on ceftriaxone and azithromycin as a treatment for community-acquired pneumonia based on her radiological findings, despite the lack of fever, leukocytosis, or elevated lactic acid.

The patient failed to respond to therapy, and her oxygen requirement increased over the following days. The patient ultimately required intubation and mechanical ventilation due to hypoxemic respiratory failure on hospital day six. Upon intubation, the patient’s oxygen saturation improved to 92-99% on a fraction of inspired oxygen of 100% and positive end-expiratory pressure of 5. Her peak airway pressure was elevated at 30-35 cmH_2_O, but it could be attributed to her obesity rather than intrinsic airway resistance. No bloody discharge was observed on initial intubation, or afterward on endotracheal tube suctioning.

COVID-19 testing was repeated multiple times, and *Mycoplasma pneumoniae* and QuantiFERON testing for tuberculosis were also performed, all yielding negative results.

The patient underwent a bronchoscopy which on irrigation of the airway returned bloody endotracheal aspirate. Blood and sputum cultures were negative, and so were the bronchoalveolar lavage aspirate and bronchial brushing cultures.

**Figure 4:**
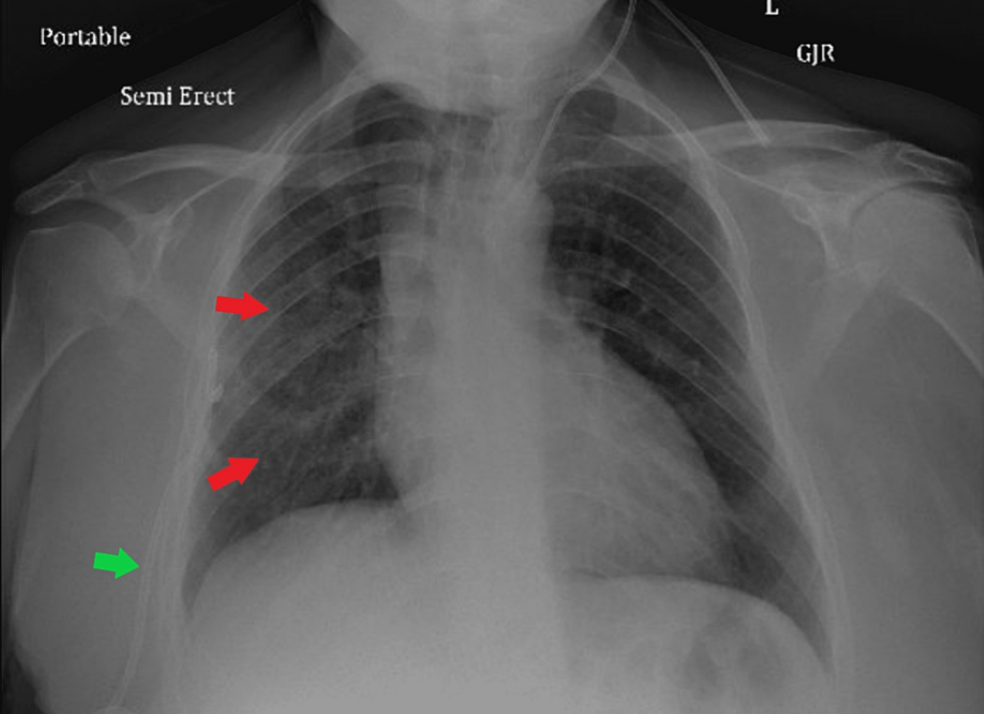
Follow-up chest X-ray revealing consistent opacities with a right-sided chest tube in place.

On the 10th hospital day, the patient had a sudden drop of hemoglobin to 6.0 mg/dL and required the transfusion of a total of four units of packed red blood cells. Haptoglobin level was found to be decreased, and reticulocyte count was increased, but direct and indirect Coomb’s tests were negative. C-reactive protein and erythrocyte sedimentation rate continued to increase at 11.5 ng/dL and 60 mm/hour, respectively. She also developed right-sided pneumothorax that necessitated the insertion of a chest tube (Figure 4).

**Figure 5:**
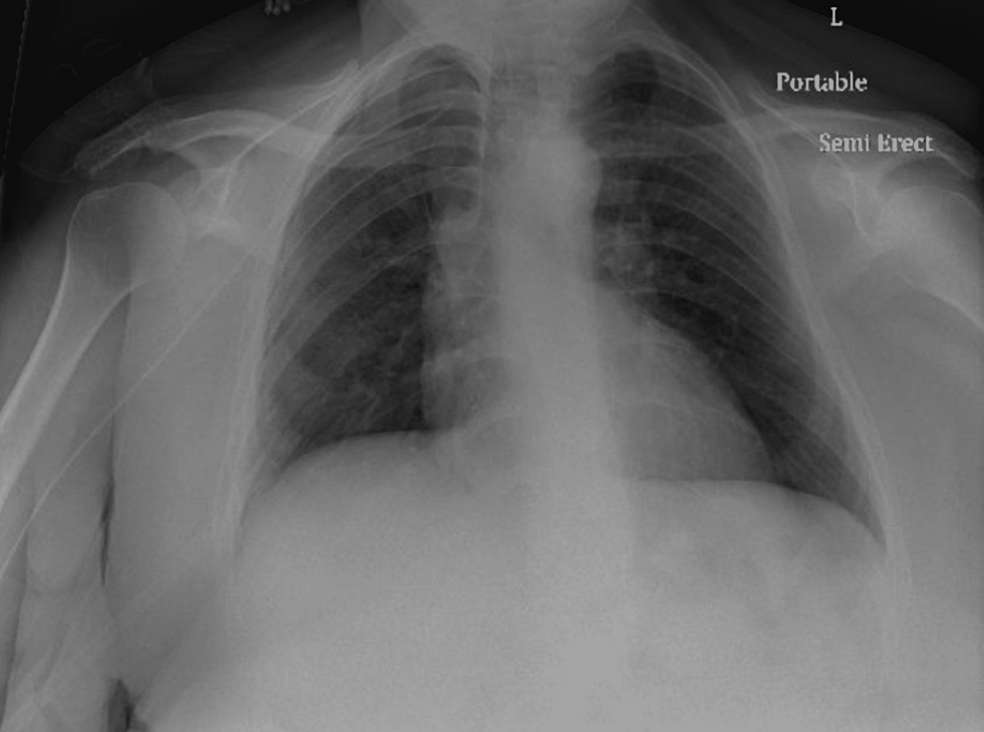
Chest X-ray prior to discharge revealing resolution of the lung opacities bilaterally.

The patient was started on high-dose steroid therapy, methylprednisone 40 mg every six hours, when suspicion of vasculitic inflammatory process was considered. The patient’s body weight was measured at around 140 kg, and the methylprednisone dose was calculated at approximately 1 mg/kg/day divided over four doses to treat for acute respiratory distress syndrome. After the initiation of steroid therapy, her inflammatory markers and oxygen requirement started to decline. Her chest X-ray immediately started to show improvement once steroids were started (Figure 5).

Autoimmune markers were ordered which revealed an elevated antinuclear antibody with a 1:80 titer and cytoplasmic/reticular antimicrobial antibody pattern, but normal complement levels, with negative anti-glomerular basement membrane antibody, negative cardiolipin antibodies, and negative Sjogren’s antibodies. c-ANCA was negative but p-ANCA was positive, with a myeloperoxidase titer of >800.

Before the autoimmune workup, the patient had undergone a video-assisted thoracic surgery biopsy. The pathology report resulted in findings consistent with ANCA vasculitis. The patient’s steroid dose was increased to pulse dosing at 250 mg every six hours before she was started on bi-weekly rituximab induction therapy at 1 g for two doses.

She was successfully extubated and subsequently discharged on a steroid taper and outpatient follow-up with rheumatology, hematology, and pulmonology.

## Discussion

ANCA-associated vasculitis can present with a myriad of differing symptoms depending on the affected vessels, but symptoms can be attributed to multiple other factors (e.g., infections, drug reactions/toxicities, neoplastic, etc.). Because vasculitis can either be a primary or a secondary disorder, ruling out possible underlying causes is important as it affects the management plan [2].

Active vasculitis, manifesting with pulmonary or renal disease, the treatment strategy is usually divided into an induction phase with high-dose steroids plus an immunosuppressive agent such as rituximab or cyclophosphamide to achieve disease remission, followed by a maintenance phase to ensure disease inactivity and prevent relapse [3].

Our patient’s abrupt onset of symptoms pointed the finger toward vasculitis as a possible cause, but respiratory tract infections had to be ruled out before considering starting therapy. Her negative leukocytic count, COVID-19 and tuberculosis testing, and blood and sputum cultures affirmed the possibility of an autoimmune process taking place.

Steroid therapy was initiated out of frustration when the patient’s condition continued to deteriorate despite proper initial management, and rituximab therapy was only considered after the hemolytic component manifested. Normally, vasculitis therapy is started as soon as the condition is suspected and infection has been ruled out. Waiting for the serum markers or pathology results would delay the treatment and put the patient at risk for complications or having a worse prognosis than had therapy been started sooner [4].

Our patient’s clinical presentation was not clear enough to suspect vasculitis, but the lack of extrapulmonary symptoms and disease progression despite adequate treatment for pneumonia raised the suspicion for vasculitis, and the patient improved dramatically after starting steroids. The delay was only secondary to the time it took to rule out infection, which is an unfortunate but necessary step to prevent inflammatory flare up by adding immunosuppressive therapy to an infectious process.

## Conclusions

The unclear presentation of vasculitis can sometimes be challenging, especially with no extrapulmonary symptoms. The delay resulting from the importance of having to rule out infection is enough burden on the patient without adding the time it would take for the suspicion of vasculitis to rise in unclear situations.

In this case presentation, we demonstrated a case where the index of suspicion was low due to the ambiguous onset which was followed by a rapid escalation of symptomatology.

We hope that the diagnosis of autoimmune vasculitis would be brought closer to the initial list of differentials of patients presenting only with pulmonary dysfunction.
